# Imaging of Treatment Response to the Combination of Carboplatin and Paclitaxel in Human Ovarian Cancer Xenograft Tumors in Mice Using FDG and FLT PET

**DOI:** 10.1371/journal.pone.0085126

**Published:** 2013-12-26

**Authors:** Mette Munk Jensen, Kamille Dumong Erichsen, Fredrik Björkling, Jacob Madsen, Peter Buhl Jensen, Maxwell Sehested, Liselotte Højgaard, Andreas Kjær

**Affiliations:** 1 Cluster for Molecular Imaging, Faculty of Health and Medical Sciences, University of Copenhagen, Copenhagen, Denmark; 2 Department of Clinical Physiology, Nuclear Medicine & PET, Rigshospitalet, Copenhagen, Denmark; 3 Topotarget A/S, Copenhagen, Denmark; 4 Department of Drug Design and Pharmacology, Faculty of Health and Medical Sciences, University of Copenhagen, Copenhagen, Denmark; Northwestern University Feinberg School of Medicine, United States of America

## Abstract

**Introduction:**

A combination of carboplatin and paclitaxel is often used as first line chemotherapy for treatment of ovarian cancer. Therefore the use of imaging biomarkers early after initiation of treatment to determine treatment sensitivity would be valuable in order to identify responders from non-responders. In this study we describe the non-invasive PET imaging of glucose uptake and cell proliferation using 2-deoxy-2-[^18^F]fluoro-D-glucose (FDG) and 3’-deoxy-3’-[^18^F]fluorothymidine (FLT) for early assessment of treatment response in a pre-clinical mouse model of human ovarian cancer treated with carboplatin and paclitaxel.

**Methods:**

*In* *vivo* uptake of FLT and FDG in human ovarian cancer xenografts in mice (A2780) was determined before treatment with carboplatin and paclitaxel (CaP) and repeatedday 1, 4 and 8 after treatment start. Tracer uptake was quantified using small animal PET/CT. Tracer uptake was compared with gene expression of Ki67, TK1, GLUT1, HK1 and HK2.

**Results:**

Tumors in the CaP group was significantly smaller than in the control group (p=0.03) on day 8. On day 4 FDG SUVmax ratio was significantly lower in the CaP group compared to the control group (105±4% vs 138±9%; p=0.002) and on day 8 the FDG SUVmax ratio was lower in the CaP compared to the control group (125±13% vs 167±13%; p=0.05). On day 1 the uptake of FLT SUVmax ratio was 89±9% in the CaP group and 109±6% in the control group; however the difference was not statistically significant (p=0.08).

**Conclusions:**

Our data suggest that both FDG and FLT PET may be used for the assessment of anti-tumor effects of a combination of carboplatin and paclitaxel in the treatment of ovarian cancer. FLT provides an early and transient signal and FDG a later and more prolonged response. This underscores the importance of optimal timing between treatment and FLT or FDG imaging since treatment response may otherwise be overlooked.

## Introduction

Ovarian cancer is the second most common gynecological malignancy and the leading cause of gynecological cancer related death in women in Europe and the Unites States [1,2]. The combination of carboplatin and paclitaxel is commonly used as first-line chemotherapy for treatment of ovarian cancer [3]. The overall response rate of carboplatin and paclitaxel therapy is 60-80% and although this response rate is relatively high compared to standard treatment of other malignancies several patients does not respond to the therapy [4,5]. In the responding patient population many patients relapse and, dependent on the time from the first treatment until relapse, a second treatment regime with platinum-based chemotherapy may be initiated. The response rate for treatment of patients with relapse is 20-30% if the platinum free interval is 6-12 months and >60% for platinum-free intervals of 12-18 months [5]. 

Evaluation of therapeutic response is frequently based on Response Evaluation Criteria in Solid Tumors (RECIST) guidelines where evaluation of treatment response is based on morphological imaging with computed tomography (CT) or magnetic resonance imaging (MRI) [6]. Anatomical imaging with CT and MRI does not provide information on the early biological processes induced by the therapy and decrease in tumor sizes is often first detectable later in the treatment course. However, early biological changes might be predictive for clinical regression before treatment effect can be assessed by anatomical imaging. Therefore, determination of tumor sensitivity early during treatment and by that identification of responders and non-responders could potentially allow for a personalized treatment approach as therapy could be modified in the non-responding patients.

Positron emission tomography (PET) imaging is a non-invasive, whole body technique where it is possible to measure physiological processes *in vivo* thereby circumventing the process of acquiring serial biopsies. Identification of a PET tracer that early after initiation of an anti-cancer treatment gives information that can predict treatment outcome is therefore of considerable interest. The comparison of tracer uptake in tumors before and in the beginning of treatment is used for monitoring the biological processes and responses evoked by the treatment. The glucose analogue 2-deoxy-2-[^18^F]fluoro-D-glucose (FDG) and the thymidine analogue 3’-deoxy-3’-[^18^F]fluorothymidine (FLT) are two of the most widely studied PET tracers used for treatment monitoring.

Imaging of metabolism with the glucose analogue FDG is used for diagnosis and staging of cancer and has high diagnostic accuracy for various tumor types. FDG crosses the cell membrane by glucose transporters whereby it is phosphorylated by intracellular hexokinases (HK) which results in intracellular trapping despite no further metabolism of the phosphorylated FDG. Glucose transporters and hexokinases are up-regulated in several cancer forms which lead to a high FDG uptake in tumor compared to normal cells [7,8].

The thymidine analogue FLT is used for imaging of cell proliferation with PET. FLT is incorporated into cells by the pyrimidine salvage pathway paralleled with thymidine and after uptake into cells FLT is phosphorylated by thymidine kinase 1 (TK1). The phosphorylation leads to intracellular trapping even though the phosphorylated FLT is not being incorporated into DNA [9]. The activity of TK1 is coupled to the cell cycle and it is mainly expressed during the S-phase [10,11]. FLT uptake is positively correlated with cell growth and TK1 activity [11-13] and in several studies a positive correlation between FLT uptake and tumor cell proliferation measured by Ki67 immunohistochemistry (IHC) is found [14-24]. 

FDG and FLT PET have in both pre-clinical and clinical studies been evaluated as imaging biomarkers that can predict and assess responses to various types of chemotherapeutic agents in several tumor types [14,19,20,22,25-36]. The results are variable, in some studies early changes in tracer uptake predict later tumor regression and in other studies no changes in tracer uptake are observed despite the treatment being effective. The mechanisms behind changes in tracer uptake after treatment initiation seem to be complex and dependent on both the tumor type and mode of action of the anti-cancer drug. In addition, the tumor baseline tracer avidity influences whether or not FDG or FLT can be used for prediction of anti-cancer treatment response e.g. in tumors with low baseline tracer avidity, decrease in tracer uptake is difficult to determine.

Carboplatin belongs to the group of platinum based anti-cancer agents causing intra-strand crosslinks in DNA which affects DNA repair and replication. Carboplatin causes cell cycle arrest in the G2 phase and induces apoptosis if the DNA damage is not properly repaired [37]. Paclitaxel binds to and stabilizes the microtubules which causes cell cycle arrest in the G2/M phase and induction of apoptotic cell death [38].

Ovarian cancer is often positive on FDG PET; however, few studies have studied prediction of ovarian cancer patient outcome after initiation of anti-cancer therapy with FDG PET [39]. In one study changes in FDG-PET uptake was predictive of patient outcome after the first cycle of neo-adjuvant chemotherapy consisting of carboplatin and paclitaxel combination therapy [40]. FDG PET was more accurate than either clinical or histopathologic response criteria or the tumor marker cancer antigen 125 (CA125) to predict treatment outcome. In another study, on the effect of neo-adjuvant treatment with carboplatin and paclitaxel in patients with ovarian cancer, it was found that in patients where the tumor uptake of FDG was equal to normal surrounding tissue uptake after 3 courses of chemotherapy these were more likely to benefit from 3 additional courses of chemotherapy than patients without normalization of FDG uptake [41]. 

Baseline FLT uptake has been analyzed in a small group of ovarian cancer patients where FLT uptake was higher in malignant compared to normal ovarian tissue [42]. In a pre-clinical study FLT uptake was decreased following effective mTOR inhibition with everolimus in a cisplatin-resistant ovarian tumor mouse model [25]. In cisplatin-sensitive ovarian cancer xenografts both FLT and FDG uptake are decreased day 4 after initiation of treatment with cisplatin [43]. However, to our knowledge, no study has yet compared changes in FLT and FDG after treatment with the combination of carboplatin and paclitaxel in a pre-clinical ovarian tumor model. Results from such a study would be clinically relevant since they could be used for selection of PET tracer and imaging time-points.

The aim of this study was therefore to determine and compare glucose uptake and cell proliferation by use of FDG and FLT PET following treatment with a combination of carboplatin and paclitaxel in a xenograft mouse model of human ovarian cancer. The FDG uptake was compared with gene expression of GLUT1, HK1 and HK2 and FLT uptake was compared with gene expression of Ki67 and TK1.

## Materials and Methods

### Tumor model

Animal care and all experimental procedures were performed under the approval of the Danish Animal Welfare Council (2006/561-1124). Eight week old female NMRI nude mice (Taconic Europe, Lille Skensved, Denmark) were used for generation of the A2780 xenograft model. All mice were acclimatized for one week in the animal facility before injection of tumor cells. The human ovarian carcinoma cell line A2780 was cultured in RPMI (Roswell Park Memorial Institute) medium 1640 + GlutaMAX (Invitrogen, Carlsbad, CA, USA) supplemented with 10% fetal calf serum (Biological Industries, Israel) and 1% penicillin-streptomycin (Invitrogen) in 5% CO_2_ at 37°C. The cell line was tested free of mycoplasma. For establishment of xenografts 10^7^ cells were diluted in 100 μL medium and mixed with 100 μL Matrixgel™ Basement Membrane Matrix (BD Biosciences, San Jose, CA, USA) for each tumor and injected into the left and right flank respectively.

### Experimental design

*In vivo* uptake of FDG and FLT in human ovarian cancer xenografts in mice was determined. Four groups of mice were used (4 mice/group). Baseline tumor sizes were approximately 100 mm^3^ on day -2. Two groups received a combination of carboplatin (Hospira, Illinois, USA) and paclitaxel (Actavis, Gentofte, Denmark) and two groups received vehicle (isotonic saline). The control groups were identical with the control groups in a previously published study as the two studies were carried out in parallel [44]. Doses were 40 mg/kg ip for carboplatin and 10 mg/kg iv for paclitaxel injected on day 0 and 5. One treatment group (n=8 tumors) and one control group (n=5 tumors) received FDG scans and one treatment group (n=6 tumors) and one control group (n=7 tumors) received FLT scans. Baseline FDG or FLT PET scans were made before treatment (day 0 or day -2) and repeated on day 1, 4 and 8 after start of treatment. Tracer uptake was in all cases quantified using small animal PET/CT. During the experiments the tumor sizes were measured by microCT [45,46]. On day 8 immediately after the last PET/CT scan all tumors were excised and gene expression of GLUT1, HK1, HK2, Ki67 and TK1 were subsequently measured by qPCR. 

### Synthesis of FDG and FLT

The radiosynthesis and quality control of FLT was performed as previously described [47]. FDG was acquired from the daily productions at Rigshospitalet (Copenhagen, Denmark).

### microPET/CT imaging

For PET imaging mice were administered approximately 10 MBq of FDG or FLT by an intravenous injection. The tracers were allowed to distribute for one hour while the animals were awake. The mice receiving FDG scans were fasted overnight before each FDG injection [48]. During the 10 minutes long PET scans the mice were anaesthetized with 3% sevofluran (Abbott Scandinavia AB, Solna, Sweden) in 35% O_2_. PET scans were acquired with a MicroPET Focus 120 (Siemens Medical Solutions, Malvern, PA, USA) and each PET scan was followed by a microCT scan acquired with a MicroCAT® II system (Siemens Medical Solutions) as previously described [47]. The mice were kept anaesthetized in the same position during the PET and CT scans allowing afterwards fusion of the images in the Inveon software (Siemens Medical Solutions). PET data were arranged into sinograms and subsequently reconstructed with the maximum a posteriori (MAP) reconstruction algorithm. The pixel size was 0.3 x 0.3 x 0.8 mm and in the center field of view the resolution was 1.2 mm full-width-at-half-maximum. The images were not corrected for attenuation or scatter. 

After fusion of PET and CT images several region of interests (ROIs) were drawn on the CT images manually by qualitative assessment covering the whole tumor in several of the tomographic planes. Thereafter all ROIs were summed and subsequently both tumor sizes and tracer uptake were calculated. The whole tumor volume defined on the CT images was therefore used for calculation of the PET tracer uptake. Tracer uptake was quantified by standardized uptake value (SUV) and the tracer uptake after treatment start was calculated relative to baseline uptake. The formula (C_T_*W)/D_inj_, where C_T_ is tissue radioactivity concentration, W is weight of the animal and D_inj_ is injected dose, was used for SUV calculations. SUVmean is a measure of the mean tissue radioactivity concentration in the tumor and SUVmax is a measure of the voxel within the ROI with the highest tracer concentration. For both SUVmean and SUVmax the uptake after treatment initiation is calculated relative to the uptake at baseline before treatment initiation and therefore the terms SUVmean ratio (SUVmean,after treatment/SUVmean,baseline) and SUVmax ratio (SUVmax,after treatment/SUVmax,baseline) were used. 

### Quantitative real-time polymerase chain reaction (qPCR)

Total RNA was isolated with TRI reagent^®^ following the manufacturer’s instructions (Molecular Research Center Inc., OH, USA). RNA concentration was determined by NanoDrop 1000 (Thermo Fisher Scientific, Wilmington, DE, USA). RNA (0.3 μg) was reversed transcribed using the Affinityscript™ QPCR cDNA Synthesis kit (Stratagene, La Jolla, CA, USA) according to the manufacturer’s instructions. 

All primers were designed in Beacon Designer (PREMIER Biosoft, Palo Alto, CA, USA). Primer sequences are shown in table 1. For each gene the primer concentrations were optimized. All samples were run in triplicate and to each sample a no-reverse transcriptional control (NoRT) was included and on each plate a no-template control (NTC) was included. 

**Table 1:** qPCR primer sequences.

Name	NCBI NM_ID	Forward primer (5’-3’)	Reverse primer (5’-3’)
GUSB	NM_000181	tgagcaagactgatacca	gctagaatagatgaccacaa
HPRT1	NM_000194	caaagcctaagatgagagt	gccacagaactagaacat
GLUT1	NM_006516	catcatcttcatcccggc	ctcctcgttgcggttgat
HK1	NM_000188	ggtgaaatcgtccgcaac	cccgggtcttcatcgtc
HK2	NM_000189	cggccgtgctacaatagg	ctcgggatcatgtgaggg
Ki67	NM_002417	tcccgcctgttttctttctgac	ctctccaaggatgatgatgctttac
TK1	NM_003258	gccgatgttctcaggaaaaagc	gcgagtgtctttggcatacttg

Gene expression was quantified on a Mx3000P® real**-**time PCR system (Stratagene) using Brilliant® SYBR® Green QPCR Master Mix (Stratagene). The thermal profile was: 10 minutes of denaturation at 95°C followed by 45 cycles of 30 seconds denaturation at 95°C, 1 minute of annealing at 60°C and 1 minute extension at 72°C. A dissociation curve was thereafter obtained by denaturation of the products for 1 minute at 95°C followed by a stepwise increase in temperature from 55°C to 95°C with steps of 0.5ºC/cycle where the duration of each cycle was 18 seconds. 

The qBase program was used for QPCR data analysis. The relative quantification of the gene of interests (GOIs) was presented as fold changes in the treatment group compared to the control group on day 8 normalized to the geometric mean of two reference genes [49]. The two most stable reference genes were found from a panel of 12 candidate genes in the human reference gene panel (TATAA Biocenter AB, Göteborg, Sweden) using the geNorm algorithm. 

### Statistical analysis

Unpaired students t-test was used for comparison between treatment and control groups. No correction for multiple comparisons was applied. Calculations were made in SPSS 20 (IBM Corporation, Armonk, New York, USA). Data are reported as mean±SEM and p<0.05 was considered statistically significant.

## Results

### Effect of carboplatin and paclitaxel treatment on A2780 tumor growth

**Figure 1:**
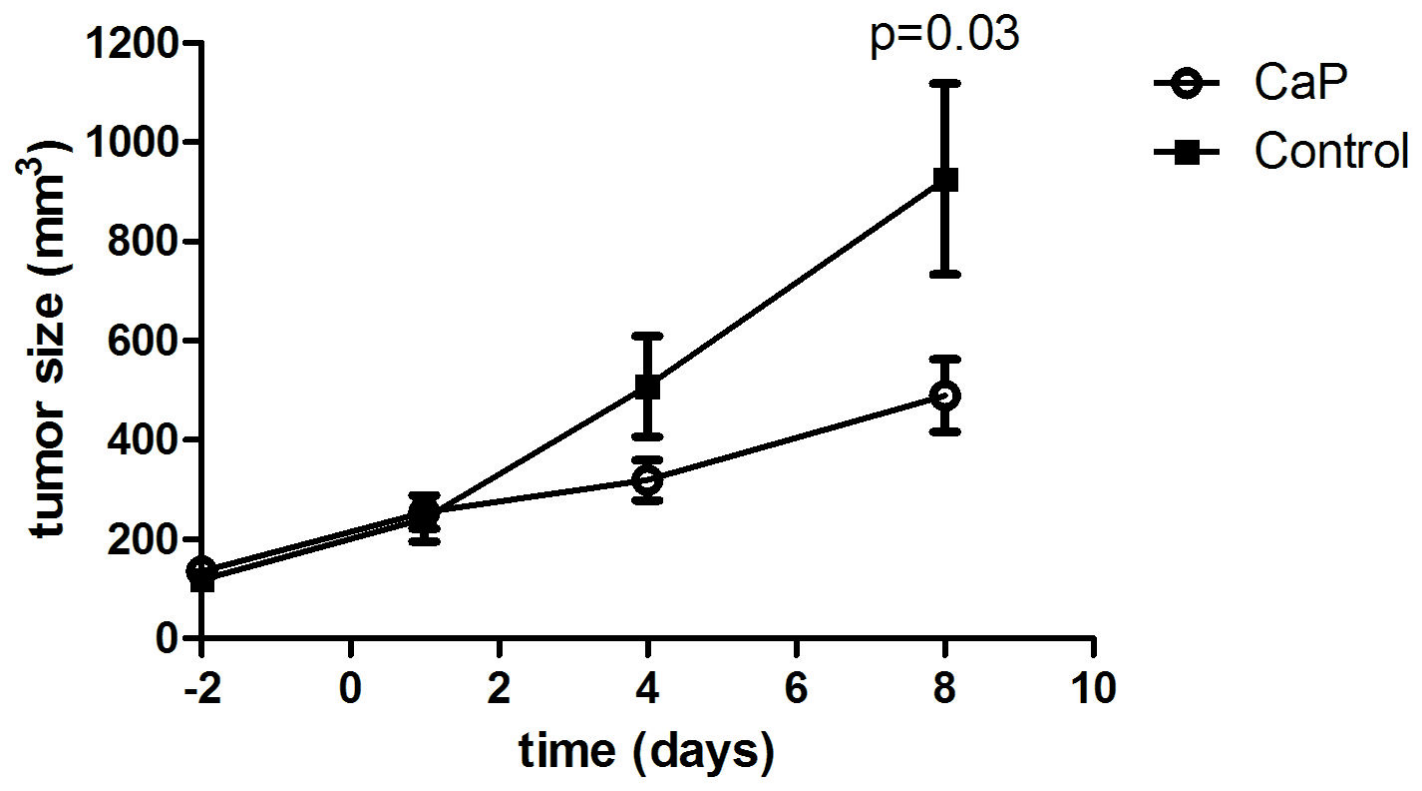
Effect of carboplatin and paclitaxel treatment on A2780 tumor growth. Mice implanted with A2780 xenograft tumors were treated with a combination of carboplatin (40 mg/kg ip) and paclitaxel (10 mg/kg iv) on day 0 and 5. Tumor sizes in the carboplatin and paclitaxel group (n=14 tumors) were significantly different than in the control group (n=12 tumors) on day 8 (p=0.034). The tumor sizes were measured with microCT and presented as mean±SEM.

Treatment of mice implanted with A2780 xenograft tumors with a combination of carboplatin (40 mg/kg ip) and paclitaxel (10 mg/kg iv) day 0 and 5 resulted in decrease in tumor size on day 8 compared to a vehicle treated control group. Baseline tumor size was 118±19 mm^3^ in the control group and 135±16 mm^3^ in the treatment group. Tumor volume in the control group was 926±192 mm^3^ on day 8 and tumors in the carboplatin and paclitaxel (CaP) group was 490±73 mm^3^ on day 8 which was significantly less than the tumors in the control group (p=0.03) (Figure 1). 

### FDG uptake in carboplatin and paclitaxel treated A2780 xenografts

**Figure 2:**
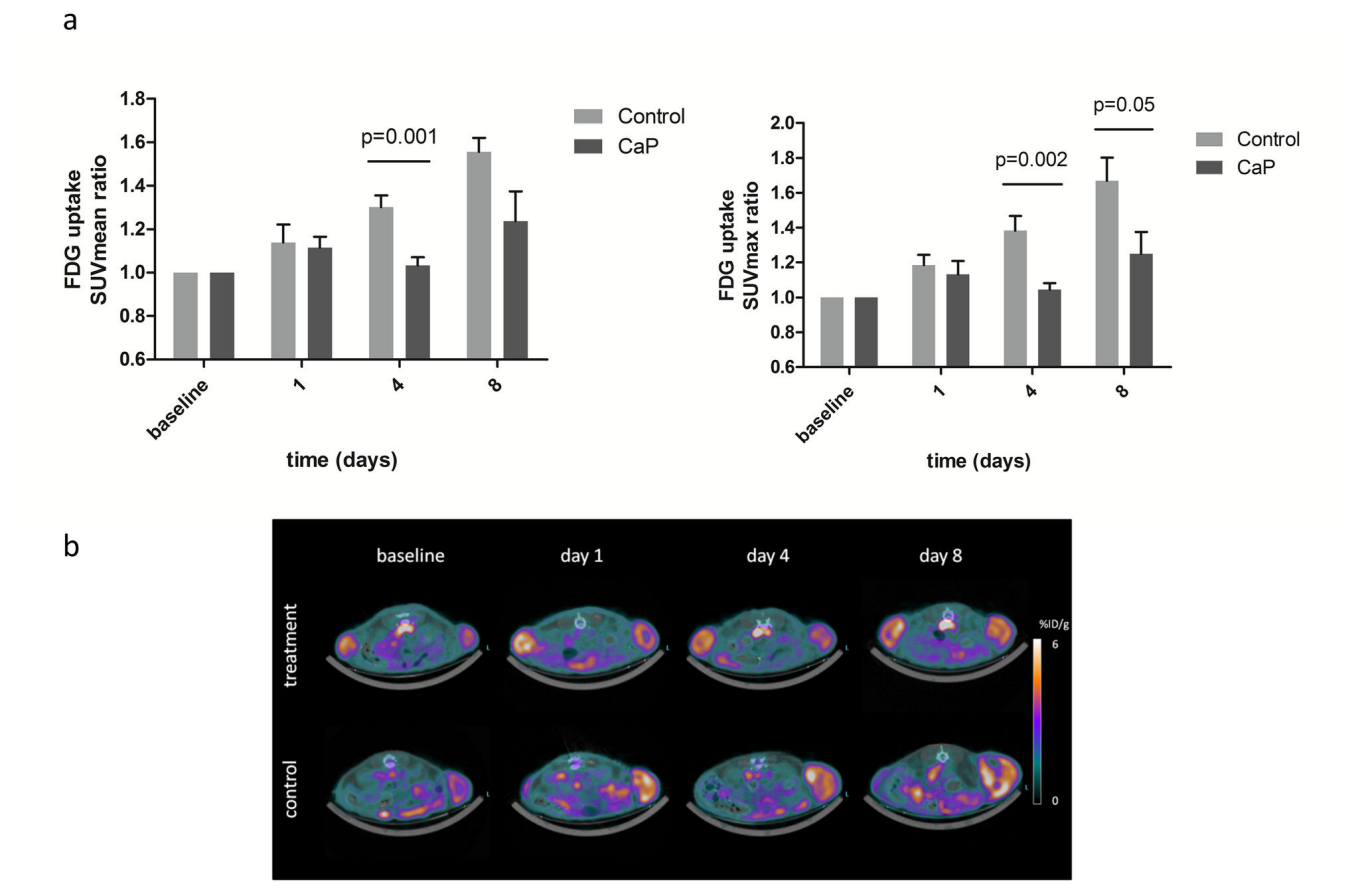
FDG uptakes after treatment with carboplatin and paclitaxel. FDG uptake was analyzed one hour post injection in a carboplatin/paclitaxel treatment (n=8 tumors) and a control group (n=5 tumors). The mice were PET/CT scanned at baseline before treatment start and day 1, 4 and 8 after injection of first dose. **A**) Tumor uptake of FDG during treatment of A2780 xenograft tumors with carboplatin and paclitaxel. Quantitative tumor uptake is presented as SUVmean and SUVmax (mean±SEM). **B**) Representative PET/CT images of one mouse from the carboplatin and paclitaxel treatment group (upper images) and one mouse from the control vehicle treated group (lower images). Dotted circles indicate the tumors.

On day 4 after initiation of treatment with carboplatin and paclitaxel FDG SUVmax ratio was significantly lower in the CaP group compared to the control group (105±4% vs 138±9%; p=0.002) and on day 8 the FDG SUVmax ratio was lower in the CaP compared to the control group (125±13% vs 167±13%; p=0.05) (Figure 2). The FDG SUVmax uptake was not significantly different between the treatment and control group at baseline and day 1 after treatment start. On day 4 FDG SUVmean ratio was significantly lower in the CaP group compared to the control group (103±4% vs 130±5%; p=0.001). The FDG SUVmean uptake was not significantly different between the treatment and control group at baseline and on day 1 and 8 after treatment start.

### FLT uptake in carboplatin and paclitaxel treated A2780 xenografts

**Figure 3:**
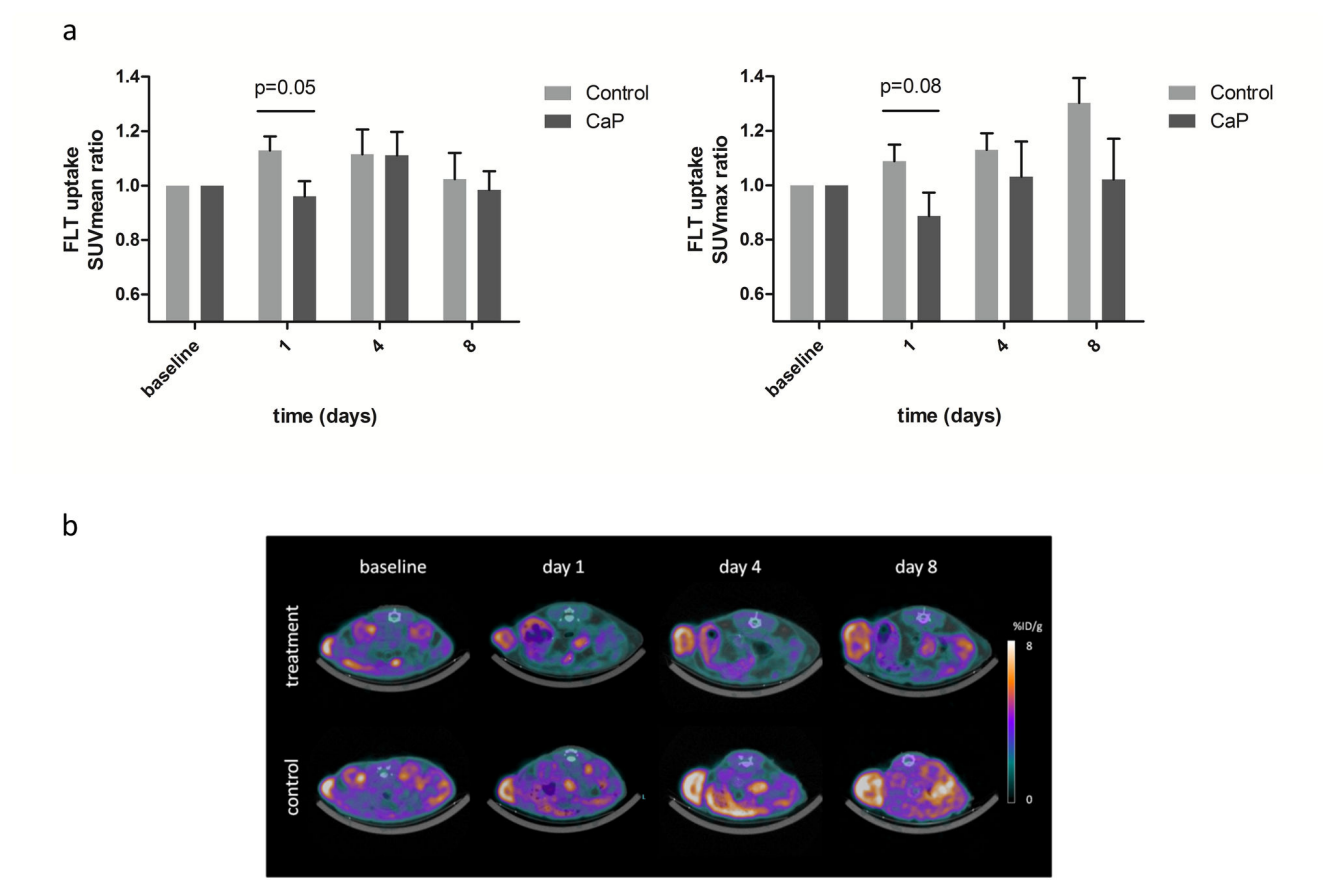
FLT uptakes after treatment with carboplatin and paclitaxel. FLT uptake was analyzed one hour post injection in a carboplatin/paclitaxel treatment (n=6 tumors) and a control group (n=7 tumors). The mice were PET/CT scanned at baseline before treatment start and day 1, 4 and 8 after injection of first dose. **A**) Tumor uptake of FLT during treatment of A2780 xenograft tumors with carboplatin and paclitaxel. Quantitative tumor uptake is presented as SUVmean and SUVmax (mean±SEM). **B**) Representative PET/CT images of one mouse from the carboplatin and paclitaxel treatment group (upper images) and one mouse from the control vehicle treated group (lower images). On the images of the mouse from the treatment group two tumors are visible whereas on the images of the control mouse only one tumor is visible. Dotted circles indicate the tumors.

On day 1 the uptake of FLT SUVmax ratio was 89±9% in the CaP group compared to 109±6% in the control group; however the difference was not statistically significant (p=0.08) (Figure 3). At all other time points the FLT SUVmax uptake in the CaP group was comparable to the control group. On day 1 the uptake of FLT SUVmean ratio was lower in the CaP compared with the control group (96±6% vs 113±5%; p=0.05). At all other time points no difference was observed between the treatment and control group.

### Gene expression of GLUT1, HK1, HK2, Ki67 and TK1

**Figure 4:**
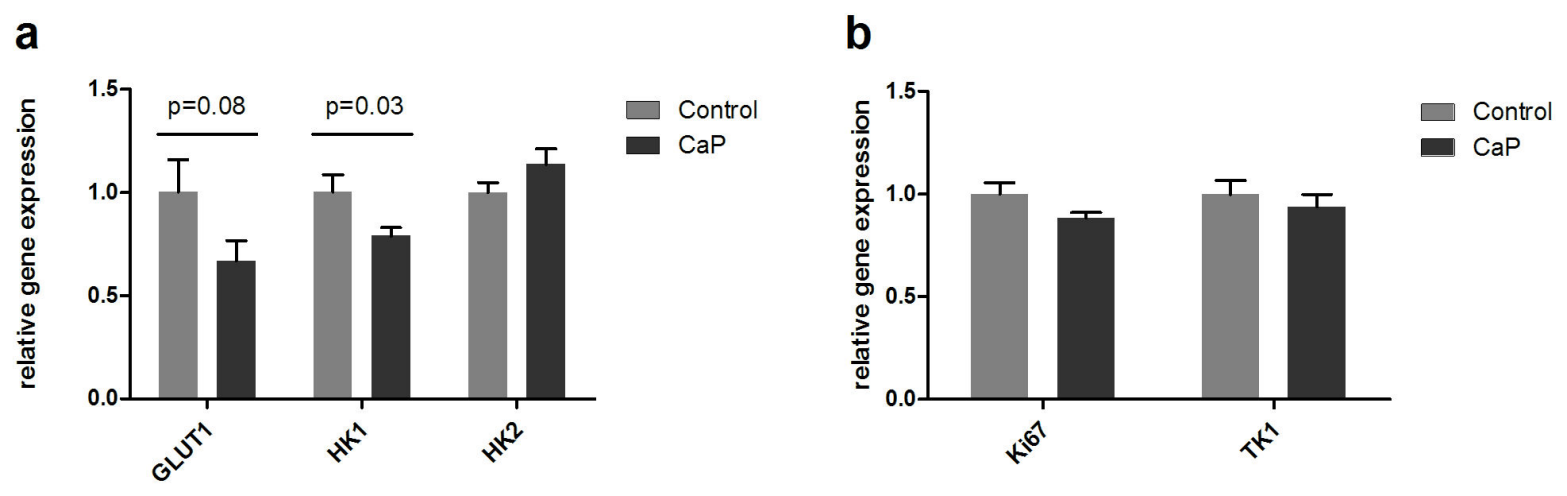
Gene expression of GLUT1, HK1, HK2, Ki67 and TK1. On day 8 immediately after the last PET/CT scan all tumors were excised and total RNA was isolated and afterward revers transcribed into cDNA. With qPCR relative expression of GLUT1, HK1, HK2, Ki67 and TK1 were measured. The levels of the gene of interests were normalized to the geometric means of two reference genes GUSB and HPRT. **A**) For the FDG study GLUT1 expression was decreased, the difference not being statistically significant (p=0.08), HK1 expression was lower in the treatment compared to the control group (p=0.03). **B**) For the FLT study no difference in Ki67 and TK1 between the treatment and control group was observed on day 8. The data are presented as mean±SEM.

The two most stably expressed reference genes were beta-glucuronidase (GUSB) and hypoxanthine phosphoribosyltransferase 1 (HPRT). The levels of the GOIs were therefore normalized to the geometric mean of GUSB and HPRT. In the treatment group GLUT1 expression was 67±10% compared to 100±16% in the control group on day 8; however, the difference was not statistically significant (p=0.08). In the treatment group HK1 expression was 79±4% compared to 100±8% in the control group on day 8 which was statistically significant (p=0.03) (Figure 4). No differences in expression of HK2, Ki67 and TK1 were observed between the treatment and control groups on day 8.

## Discussion

In this study we describe the non-invasive imaging of glucose uptake and cell proliferation for early assessment of treatment response in a pre-clinical mouse model of human ovarian cancer treated with a combination of carboplatin and paclitaxel. Glucose uptake and cell proliferation were visualized by FDG and FLT PET respectively. The A2780 cell line was used for generation of xenograft tumors in mice. This tumor model responded well to the combination therapy with carboplatin and paclitaxel measured as a reduction in tumor size as compared with a control group on day 8 after start of treatment. 

On day 4 after start of treatment with carboplatin and paclitaxel, uptake of FDG was significantly lower in the treatment compared to the control group and uptake remained low on day 8. The decrease in FDG uptake was paralleled by decreases in GLUT1 and HK1 expression, whereas no change in HK2 expression was observed suggesting that GLUT1 and HK1 are involved in the FDG uptake in this tumor model.

We observed an early but transient decrease in cell proliferation measured by FLT PET after treatment initiation of responding A2780 tumors with the combination of carboplatin and paclitaxel. A difference in FLT uptake between the treatment and control group was already observed on day 1 after treatment start. In contrast, FLT uptake was not different between the treatment and control mice on day 4 and 8 after treatment start. The identical cell proliferation in the treatment and control group measured by FLT PET on day 8 was supported by gene expression measurements of Ki67 and TK1 which showed no difference in Ki67 and TK1 expression between the treatment and control group on day 8. In other studies, no change in FLT uptake, despite treatment with an effective anti-cancer therapy, has also been observed [29,31]. The unchanged FLT uptake late in the treatment course could be due to several factors. The anti-cancer treatment may result in feedback activation of the *de novo* pathway of DNA synthesis resulting in unchanged or even increased FLT uptake despite decreased cell proliferation. This phenomenon has for example been observed during treatment with 5-FU [35]. The relation between the pyrimidine salvage pathway and the *de novo* pathway of DNA synthesis in tumors has a great influence on the uptake of FLT and different tumors have variable contributions of the two pathways [50,51]. Some tumors rely mainly on *de novo* synthesis of DNA precursors which will result in low baseline FLT uptake despite high cell proliferation rate [9]. Therefore it is possible that treatment of tumors relying primarily on *de novo* synthesis not necessarily will result in decreases in FLT uptake despite treatment with effective chemotherapy that reduces cell proliferation [31]. Previously, we have reported that effective anti-cancer treatment reduced FLT uptake in the A2780 human ovarian cancer xenograft mouse model indicating that the salvage pathway contributes to the thymidine requirement in this tumor model [46,47,52]. No decrease in the cell proliferation associated genes Ki67 and TK1 were observed on day 8 despite effective treatment. Thus, it seems likely that the combined treatment with carboplatin and paclitaxel does not change the cell proliferation late in the treatment course even though the therapy reduces the tumor growth. Carboplatin and paclitaxel were administered by injection on day 0 and 5. PET imaging was performed on day 4 and 8 and thus tracer uptake was measured on day 4 after the 1^st^ and on day 3 after the 2^nd^ administration of chemotherapy. This may suggest that the treatment does decrease cell proliferation for less than 3 days and this is the reason why no difference in cell proliferation was observed on day 4 and 8 because the tumor cells has started to re-proliferate. Further investigations are needed to determine for how long the proliferation is affected after a single injection of carboplatin and paclitaxel. 

Both carboplatin and paclitaxel cause cell cycle arrest in the G2/M phase. Phosphorylation of FLT by TK1 is assumed to be the limiting factor for FLT uptake and activity of TK1 is correlated with FLT uptake [13]. TK1 is mainly expressed in the S-phase of cell cycle, therefore cell cycle arrest later in the cell cycle might not influence uptake of FLT.

The differences between cell proliferation and glucose uptake after initiation of carboplatin and paclitaxel therapy illustrates the importance of combining imaging of several physiological processes in order to analyze the biological effect of cancer treatment. Carboplatin and paclitaxel treatment induced significant decreases in FDG uptake after day 4 and onwards but was less effective in reducing tumor cell proliferation as measured by FLT. However FLT was an earlier marker than FDG, although transient. Also this underscores the importance of optimal timing between treatment and FLT or FDG imaging since treatment response may otherwise be overlooked. Thus, in order to find an imaging biomarker that potentially could be predictive for treatment outcome in future clinical studies different imaging biomarkers, giving information of different physiological processes, need to be evaluated.

One of the main limitations of the present study was that a limited number of animals were included in each group. This could be the reason that several of the measurements did not reach statistical significance due to type II error. Furthermore does the present study describe treatment response monitoring in human xenograft tumors in nude mice. Although the tumors are of human origin, the non-human host environment may cause that results acquired in this pre-clinical model not necessarily can be translated into clinical studies. Another limitation of the study was that the molecular markers were measured on the gene expression level and it is therefore unknown whether or not the gene expression levels are a reflection of the protein expression. 

A future application of treatment response monitoring with FDG and FLT for ovarian cancer could potentially be evaluation of treatment effect in protocols evaluating the effect of adding e.g. targeted anti-cancer agents to the carboplatin and paclitaxel standard therapy. Future studies are needed to determine if FDG and FLT PET are applicable for prediction and monitoring the outcome of supplementary combination in comparison with a standard treatment.

In conclusion, we found that the change of FDG and FLT uptake after initiation of therapy with carboplatin and paclitaxel were different but complementary. FLT provides an early and transient signal and FDG a later and more prolonged response. Thus, our data suggest that both FDG and FLT PET may be used for the assessment of anti-tumor effects of a combination of carboplatin and paclitaxel in the treatment of ovarian cancer. 
